# IGF-IR Promotes Prostate Cancer Growth by Stabilizing α_5_β_1_ Integrin Protein Levels

**DOI:** 10.1371/journal.pone.0076513

**Published:** 2013-10-09

**Authors:** Aejaz Sayeed, Carmine Fedele, Marco Trerotola, Kirat K. Ganguly, Lucia R. Languino

**Affiliations:** Department of Cancer Biology, Prostate Cancer Discovery and Development Program, Thomas Jefferson University, Philadelphia, Pennsylvania, United States of America; University of Kentucky College of Medicine, United States of America

## Abstract

Dynamic crosstalk between growth factor receptors, cell adhesion molecules and extracellular matrix is essential for cancer cell migration and invasion. Integrins are transmembrane receptors that bind extracellular matrix proteins and enable cell adhesion and cytoskeletal organization. They also mediate signal transduction to regulate cell proliferation and survival. The type 1 insulin-like growth factor receptor (IGF-IR) mediates tumor cell growth, adhesion and inhibition of apoptosis in several types of cancer. We have previously demonstrated that β_1_ integrins regulate anchorage-independent growth of prostate cancer (PrCa) cells by regulating IGF-IR expression and androgen receptor-mediated transcriptional functions. Furthermore, we have recently reported that IGF-IR regulates the expression of β_1_ integrins in PrCa cells. We have dissected the mechanism through which IGF-IR regulates β_1_ integrin expression in PrCa. Here we report that IGF-IR is crucial for PrCa cell growth and that β_1_ integrins contribute to the regulation of proliferation by IGF-IR. We demonstrate that β_1_ integrin regulation by IGF-IR does not occur at the mRNA level. Exogenous expression of a CD4 - β_1_ integrin cytoplasmic domain chimera does not interfere with such regulation and fails to stabilize β_1_ integrin expression in the absence of IGF-IR. This appears to be due to the lack of interaction between the β_1_ cytoplasmic domain and IGF-IR. We demonstrate that IGF-IR stabilizes the β_1_ subunit by protecting it from proteasomal degradation. The α_5_ subunit, one of the binding partners of β_1_, is also downregulated along with β_1_ upon IGF-IR knockdown while no change is observed in the expression of the α_2,_ α_3,_ α_4,_ α_6_ and α_7_ subunits. Our results reveal a crucial mechanistic role for the α_5_β_1_ integrin, downstream of IGF-IR, in regulating cancer growth.

## Introduction

Adhesion of cells to extracellular matrix (ECM) is primarily mediated by integrins and is crucial for cell growth and survival. Integrins are heterodimeric transmembrane receptors, consisting of α and β subunits, that are non-covalently associated; they physically link the ECM to the intracellular actin cytoskeleton but are also able to transduce signals bidirectionally across the plasma membrane [1]. By binding to ECM ligands, integrins are activated and able to regulate cellular functions by initiating intracellular cascades of signaling. So far, 24 integrin heterodimers, 18 α and 8 β subunits and five β_1_variantsubunits β_1_Aβ_1_Bβ_1_Cβ_1_C-2 and β_1_D, generated by alternative splicing, have been described [2] [3]. Integrins are critical regulators of growth, differentiation, survival, migration and invasion [4], [5]. It has been reported that progression of prostate cancer (PrCa) to advanced stages is associated with changes in integrin expression profiles [6], [7], [8].

The pathways of integrin and growth factor signaling are thought to be mechanistically linked because cell adhesion to ECM is crucial for cells to respond to certain growth factors [9]. Growth factor signaling can disrupt focal adhesions, the presumed sites of integrin-mediated signaling [9] and consequently modulate integrin-mediated cell adhesion and motility. Physical and functional interactions between integrins and components of growth factor signaling pathways, including insulin-like growth factor 1 (IGF-1) or its downstream signaling proteins [10], [11], have been reported. Our laboratory has demonstrated that β_1_ integrins selectively modulate type 1 insulin-like growth factor receptor (IGF-IR)-mediated signaling and functions in PrCa [10], [12]. IGF-1 has also been reported to induce adhesion and migration in human multiple myeloma cells partly via activation of β_1_ integrins [13]. Furthermore, constitutively active β_1_ integrins promote malignant phenotype in PrCa cells and targeting them was reported to inhibit PrCa metastasis [14].

IGF-1 is a single chain polypeptide that in addition to its more classical endocrine role, mediates autocrine or paracrine growth and thus acts as a potent growth and survival factor. IGF-1 elicits its actions on cells by binding to its receptor, IGF-IR. The IGF-IR is a heterotetrameric transmembrane glycoprotein with tyrosine kinase activity [15]. The insulin receptor substrate (IRS) proteins function as specific docking proteins for IGF-IR and insulin receptor (IR) [16]. IRS1 and IRS2 do not contain intrinsic kinase activity but rather function by recruiting proteins to surface receptors, where they assemble signaling complexes. Signaling from the IRS proteins results in the activation of pathways including phosphatidyl inositol-3 kinase (PI3K) and mitogen-activated protein kinase (MAPK) [17], [18]. Interestingly, both pathways are also known to be activated by integrin engagement [19], [20]. The association between integrins and IRS1 has been suggested as a possible mechanism for synergistic action of growth factor and extracellular matrix receptors [21]. IGF-1 signaling has been reported to be regulated by a negative feedback mechanism via ubiquitin/proteasome mediated degradation of IRS2, whereby the magnitude and duration of the response to insulin or IGF-1 is regulated [22]. Our laboratory has recently demonstrated that β_1_ integrins regulate IGF-IR expression and are critical for IGF-1-mediated enhancement of androgen receptor (AR) activity [23]. We have also reported that IGF-IR tightly regulates β_1_ integrin expression in PrCa cells [24] but the mechanism underlying this regulation is not yet characterized.

Despite the limited consensus regarding the levels of IGF-IR expression in benign and malignant prostate epithelium, several clinical trials targeting the IGF-IR in different tumors, including PrCa, are underway. Identifying and understanding the downstream effectors of IGF-IR would help in better defining the functional role of the IGF-1 axis in PrCa. Given the reported evidence of strong physical and functional interaction between β_1_ integrins and IGF-IR, this study investigated the mechanism through which IGF-IR regulates β_1_ integrins. We report a novel pathway of crosstalk between IGF-IR and β_1_ integrins, which promotes cancer cell proliferation, and demonstrate that IGF-IR stabilizes α_5_β_1_ integrin by protecting it from proteasomal degradation.

## Materials and Methods

### Reagents and antibodies

The following reagents were used. Opti-Mem and oligofectamine (all from Invitrogen, CA), synthetic androgen R1881 (Perkin-Elmer, CA), proteinase inhibitors (Sigma, MO), recombinant IGF-1 (R&D Systems, MN), MG132 and epoxomicin (Sigma, MO). The following murine monoclonal antibodies (mAbs) were used: to β_1_ integrins (BD Transduction Laboratories, CA), to IGF-IR for flow cytometry (αIR-3, EMD, NJ); to α_2_ integrin (Abcam, Cambridge, UK); to α_7_ integrin (8G2, EMD, NJ). Rat mAb to CD4 was purchased from Santa Cruz, CA. The following rabbit polyclonal antibodies (pAbs) were used: to IGF-IR (IGF-IR-β sc713), to AKT and to ERK1/2 (from Santa Cruz, CA); to survivin (Novus Biologicals, CO). Rabbit pAbs to α_3_, α_4_, and α_5_ specific for the C-terminus domain of each subunit, were a kind gift from Dr. E. Ruoslahti, University of California Santa Barbara, Sanford-Burnham Medical Research Institute, CA. The α_6_ Ab (AA6A) specific for the C-terminal domain of human α_6_ integrin was a kind gift from Dr. Anne Cress, University of Arizona, AZ. SiRNA oligonucleotides used in this report have been described before [24].

### Cells

LNCaP and C4-2B cells were purchased from ATCC. Cells were grown at 37°C and 5% CO_2_ in RPMI-1640 supplemented with 5% FBS and 1% each of sodium pyruvate, HEPES and non-essential amino acids. To evaluate the effect of agonists, after transfection cells were starved with 2% charcoal-stripped serum (CSS) containing medium for 24 h followed by ligand stimulation for additional 24 h. PC3 cells were grown at 37°C and 5% CO_2_ in RPMI-1640 supplemented with 10% FBS. PC3-Ch1, PC3-Ch2 and PC3-Ch β_1_C cells used for inducible expression of chimeric constructs have been described earlier [25], [26]. Cells were serum-starved for 24 h and treated with 75 μM ZnSO_4_ for 6 h. The Ch1 chimeric construct contains the extracellular domain of murine CD4 and the transmembrane and cytoplasmic domains of the β_1_A integrin; Ch β_1_C construct (used as a control) is same as Ch1 except that β_1_A integrin-coding region is replaced by β_1_C coding region. Ch2 construct represents another control and carries the extracellular domain of murine CD4 joined to the transmembrane domain of the β_1_ integrin subunit. All the constructs are expressed under the control of the mouse metallothionein-1 promoter and the expression of chimeric variants is induced upon addition of ZnSO_4_ to the growth medium.

### Transient siRNA transfection

Transient transfection of cells with siRNA oligonucleotides was performed as described [23]. Inverted-IGF-IR siRNA having an inverse target sequence of IGF-IR siRNA served as control.

### Proliferation assay

LNCaP and C4-2B cells were transiently transfected with control or IGF-IR siRNA. Twenty four h post-transfection, cells were trypsinized and analyzed for the efficiency of IGF-IR and β_1_ integrin downregulation. Transfected cells were counted and re-plated in triplicates in 6-well plates at 3×10^4^ cells per well in 2% CSS-containing medium in presence of 1 nM R1881. Live cells were counted for next three consecutive days by haemocytometer. Pictures of live cells were taken on day 2 and 3 before being harvested for counting.

### Anchorage-independent growth assay

LNCaP cells were plated and transfected with control or IGF-IR siRNA in combination with either vector alone, pBJ1, or a pBJ1-β_1_ construct [27]. Twenty four h later, cells were trypsinized and plated in soft-agar in 6-well dishes at 5,000 cells/well. The cells were allowed to grow for two weeks and colonies counted. The colony size was measured by using an eyepiece equipped with a measuring reticle and colonies with size of 0.1 mm were counted in different samples. The colonies were fixed and stained with crystal violet and images of colonies were captured by stereo microscope.

### Immunoprecipitation and Immunoblotting

Immunoprecipitation of PC3 cells was carried out as described earlier [28]. Cell lysates were used for immunoblotting as described [12]. To analyze PC3 cell lysates transfected with chimeric constructs, PC3-Ch1 and PC3-Ch2 cells were transfected with control or IGF-IR siRNA and 24 h later, cells were grown in serum-free medium for 24 h followed by treatment with 75 μM ZnSO_4_ for 6 h and then, harvested for immunoblotting. The intensity of each band was evaluated by ImageJ analysis and normalized with loading control.

### FACS analysis

PC3-Ch1 and PC3-Ch2 cells were treated as above and harvested for FACS analysis. The cells were stained with 1 μg/ml Ab to CD4 or rat IgG as negative control, followed by staining with FITC-conjugated secondary Ab. Expression profiles were acquired using FACS Calibur instrument (BD) and data were analyzed by Flowjo software (Tree Star Inc., OR).

### Proteasomal inhibition assay

LNCaP cells were transfected with control or IGF-IR siRNA and 24 h later, cells were starved in 2% CSS-containing medium for 24 h. Cells were treated with 1 nM R1881 with or without 10 μM MG132 for 6 h. PC3-2 cells were transfected in the same manner as LNCaP cells and 24 h after transfection treated with 10 μM MG132 for either 6 or 24 h and analyzed by immunoblotting. For specific inhibition of the proteasome function using epoxomicin, LNCaP cells were transfected as above, starved with 2% CSS-containing medium for 24 h, followed by treatment with 1 nM R1881 together with 0, 100, 250 or 500 nM epoxomicin for 18 h and harvested. Lysates were analyzed by immunoblotting. Relative band intensities of β_1_ integrin subunits

### Quantitative real time PCR

Real time PCR analysis was performed as described earlier [23]. Each reaction was carried out, at least in triplicate; standard deviations and significance were calculated using Excel (Microsoft) software. The sequences of oligos used are as follows: β_1_ integrin, (sense: CTCAAGCCAGAGGATATTAC, antisense: TCATTGAGTAAGACAGGTCC), IGF-IR, sense: AATGAGTGCTGCCACCCCGA, antisense: ACACAGCGCCAGCCCTCAAA), GAPDH, (sense:GGGAAGGTGAAGGTCGGAGT, antisense: GTTCTCAGCCTTGACGGTGC), β-actin, (sense: TCCATCATGAAGTGTGACGT, antisense: GGAGGAGCAATGATCTTGAT).

### Statistical analysis

Statistical significance (P value and t-test) between datasets was calculated using Excel (Microsoft) software. A two-sided P value of ≤0.02 was considered statistically significant. The results were plotted on a graph using DeltaGraph 4.5 (RockWare) software.

## Results

### Loss of IGF-IR and β_1_ integrins inhibits proliferation of PrCa cells

**Figure 1:**
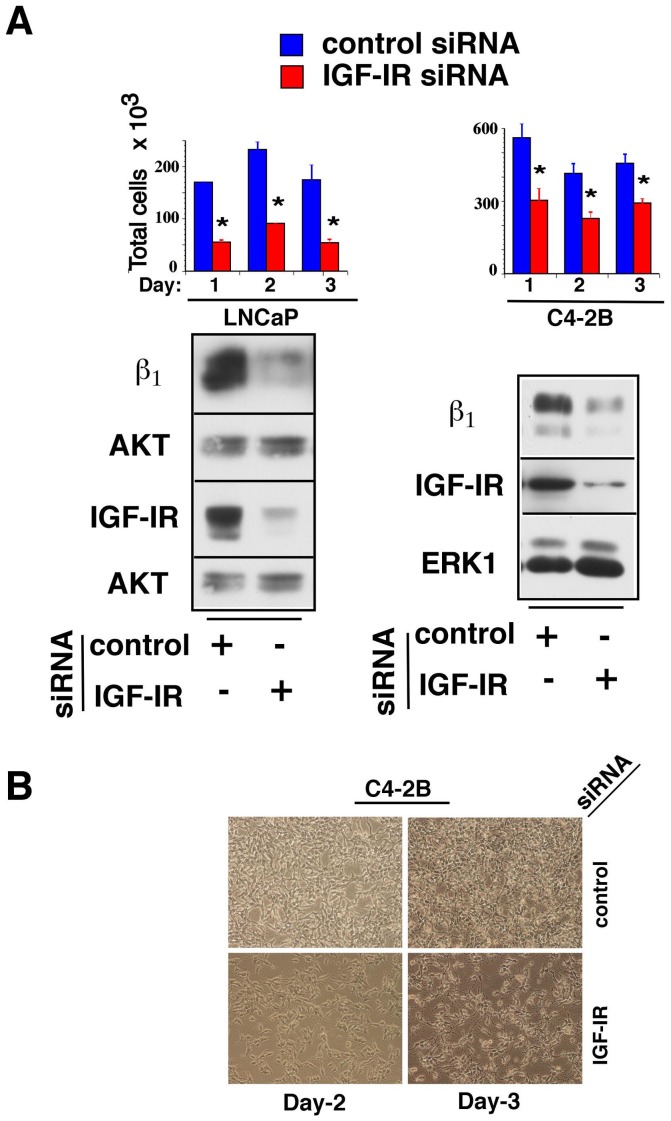
Loss of IGF-IR and β_1_ integrins inhibits proliferation of PrCa cells. (A) LNCaP and C4-2B cells were transfected with either control siRNA or IGF-IR siRNA and 24 h later, cells were trypsinized and counted. Cells were replated in triplicates at 3×10^5^ cells per well in 6-well plates with 2% CSS-containing medium in the presence of 1 nM R1881, harvested and counted at day 1, 2 and 3 after re-plating. Each experimental assay was carried out in triplicates and error bars represent standard deviation from three independent values (^*^P<0.01), relative to respective control siRNA treatments. A parallel set of LNCaP and C4-2B cell lysates was analyzed for efficiency of IGF-IR and β_1_ integrin subunit downregulation by immunoblotting (lower panels). (B) Representative images of relative C4-2B cell densities on day 2 and day 3 are shown.

We have previously demonstrated that β_1_ integrins are crucial for IGF-IR-mediated c ancer cell proliferation [10]. Since IGF-IR tightly regulates β_1_ integrin expression, we evaluated the direct effect of IGF-IR depletion on cell proliferation. LNCaP and C4-2B cells were transiently depleted of IGF-IR and re-plated in 2% CSS-containing medium in the presence of 1 nM synthetic androgen (R1881). Loss of IGF-IR strikingly inhibits cell proliferation in both cell lines (^*^P<0.02) (Fig. 1A, top panels). Reduced expression of IGF-IR and β_1_ integrin subunits for both cell lines was confirmed by immunoblotting (Fig. 1A, lower panels). R1881 was used to enhance the expression levels of IGF-IR and β_1_ and to augment the effects of these receptors on proliferation. Significant effects on cell proliferation were also observed in the absence of R1881 in LNCaP and C4-2B cells after IGF-IR depletion (data not shown). Reduced cell density in culture conditions is clearly observed upon analysis of C4-2B cells with reduced IGF-IR and β_1_ levels compared to cells with endogenous expression of both receptors (Fig. 1B). Representative cell density images of day 2 and day 3 proliferation assays are shown. These data show that IGF-IR and β_1_ integrins are essential for proliferation of PrCa cells.

### Exogenous expression of the β_1_ integrin subunit restores the impaired anchorage-independent growth of PrCa cells upon IGF-IR downregulation

**Figure 2:**
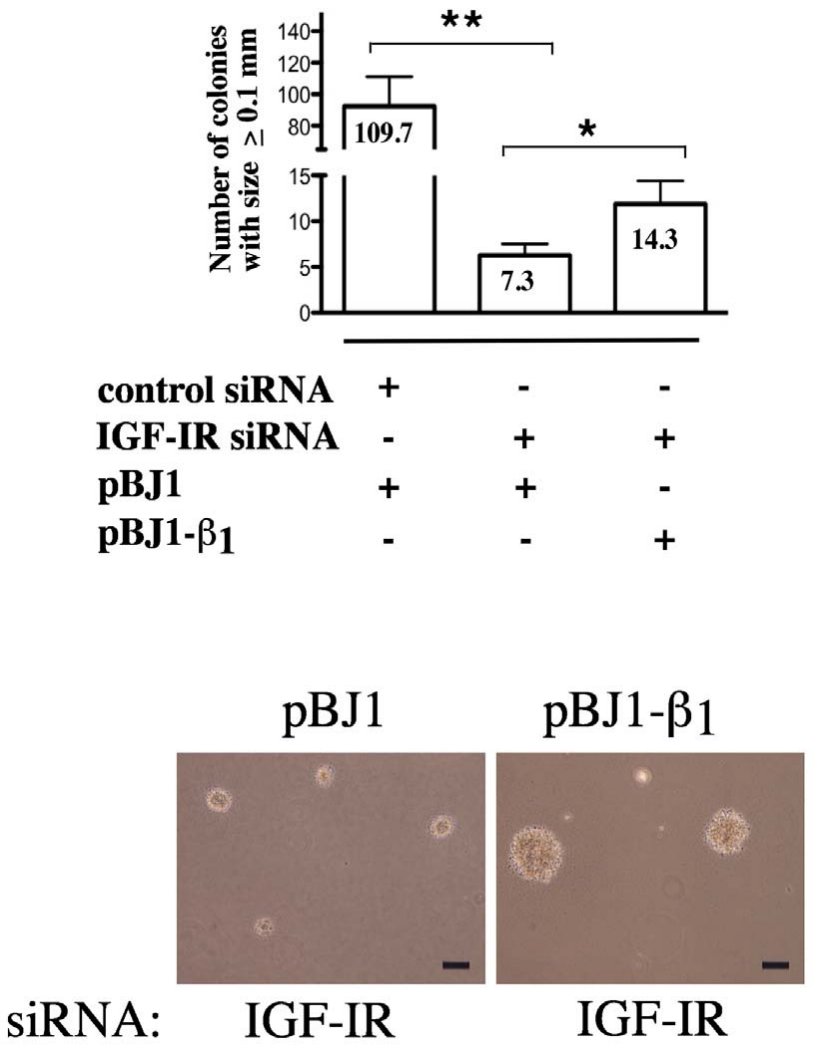
Exogenous expression of β_1_ integrins rescues the impaired anchorage-independent growth in absence of IGF-IR. LNCaP cells were co-transfected with either control or IGF-IR siRNA and with either the pBJ1-β_1_ construct or the control vector pBJ1. Cells were plated in soft-agar and allowed to grow for 2 weeks. The size of the colonies was measured using an inverted microscope equipped with an eyepiece containing a 25 mm reticle and total colonies with the size ≥100 μm were counted. The numbers shown in the graph represent the average counts from three independent samples (^*^P<0.02; ^**^P<0.001). Representative images of live colonies reflecting variation in colony size with or without exogenous β_1_ are shown in the lower panels. The measuring bars represent a size of 100 μm.

The findings that IGF-IR regulates β_1_ integrin expression and that abrogation of IGF- IR compromised the growth of cancer cells, prompted us to investigate whether β_1_ integrins play a role in IGF-IR-mediated growth regulation. In order to determine if β_1_ integrin expression would reverse the inhibition of anchorage-independent growth induced by IGF-IR depletion, LNCaP cells were transfected with IGF-IR siRNA, with or without β_1_ integrin cDNA, and allowed to grow and form colonies in soft-agar for two weeks. IGF-IR depletion significantly reduces the growth of colonies in soft-agar (^**^P<0.01) (Fig. 2). Exogenous expression of the β_1_ subunit however, partially alleviates the growth suppression induced by IGF-IR knockdown as measured by the number of colonies with size ≥100 μm (^*^P<0.02). Representative images of live colonies were captured on an inverted microscope and are shown in the lower panel (Fig. 2). These data underscore the role of β_1_ integrins in IGF-IR-mediated regulation of growth in PrCa cells.

### IGF-IR regulation of β_1_ integrin expression does not occur at the mRNA level

**Figure 3:**
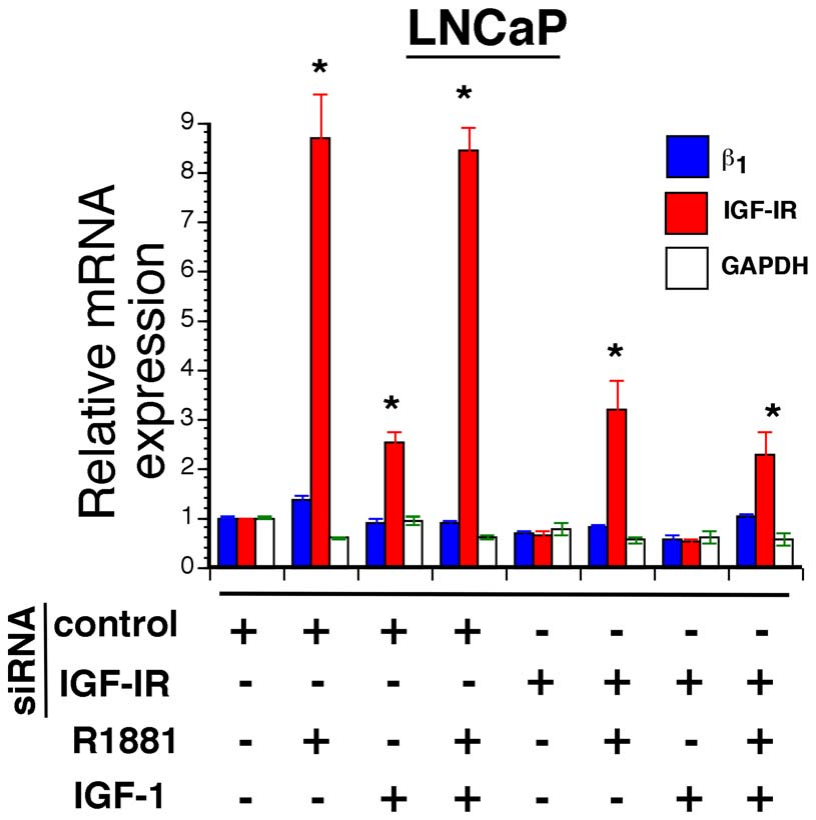
IGF-IR-mediated regulation of β_1_ integrin expression does not occur at the mRNA level. LNCaP cells were transfected with either control or IGF-IR siRNA. Twenty four h later, cells were grown in medium containing 2% CSS for additional 24 h and treated with vehicle, 1 nM R1881 or 100 ng/ml IGF-1 for additional 24 h. RNA isolated from these cells was evaluated for transcript levels of IGF-IR, β_1_ integrin and GAPDH using quantitative real time PCR. Expression values were normalized over transcript levels of β-actin and the data are presented as relative expression. Each reaction was run in triplicate and error bars represent standard deviation (^*^P<0.01) relative to untreated samples.

Cooperative effect of these receptors on growth of cancer cells led us to investigate the mechanism by which IGF-IR regulates β_1_ expression. We have demonstrated earlier that IGF-IR regulates the expression of β_1_ integrin subunits in PrCa cells [24]. This regulation may occur at the transcriptional, post-transcriptional, translational or post-translational levels. mRNA regulation of β_1_ integrins by IGF-IR was analyzed in LNCaP cells by reducing the expression of IGF-IR by RNA interference, followed by treatment with R1881 and/or IGF-1. Real time analyses of mRNA transcripts indicate that IGF-IR mRNA is induced 8-fold upon R1881 and 2.5-fold upon IGF-1 treatment (Fig. 3). However, no significant changes in β_1_ integrin mRNA levels are detected upon both treatments. Depletion of IGF-IR expression results in four-fold reduction of IGF-IR mRNA after ligand treatments. The data indicate that β_1_ integrin transcript levels do not change significantly upon IGF-IR knockdown. Analysis of GAPDH expression profile in this experiment served as an additional reference control. The results clearly indicate that IGF-IR does not regulate β_1_ integrin subunits at the mRNA level.

### Inducible expression of CD4-β_1A_ integrin cytoplasmic domain chimera does not protect the endogenous β_1_ integrin subunit from degradation induced by IGF-IR depletion

**Figure 4:**
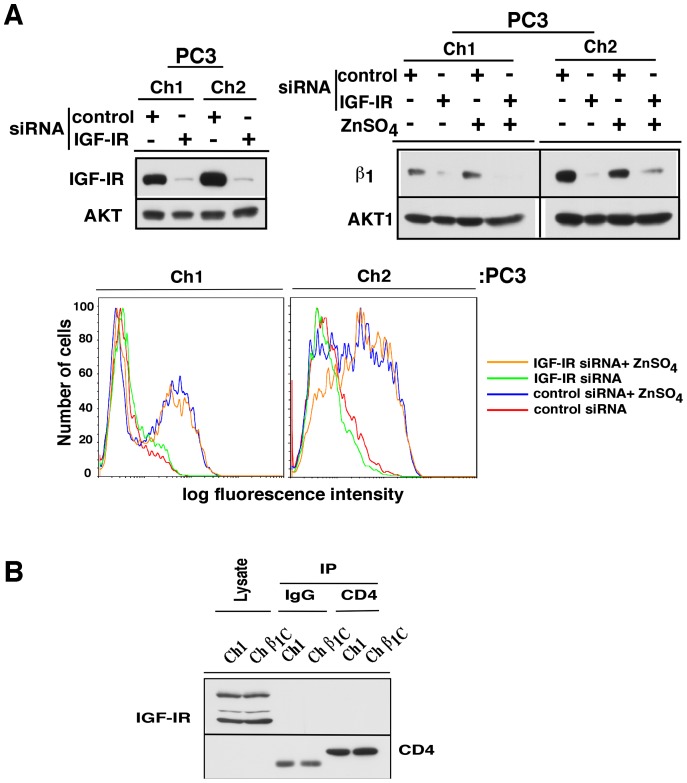
Exogenous CD4 - β_1_ integrin cytoplasmic domain chimera does not influence the IGF-IR-mediated regulation of the endogenous β_1_. (A) PC3-Ch1 (expressing extracellular domain of murine CD4 and the transmembrane and cytoplasmic domains of β_1_) and control PC3-Ch2 cells (expressing the extracellular domain of murine CD4 joined to the transmembrane domain of the β_1_) were transfected with either control or IGF-IR siRNA. Twenty-four h post transfection, cells were harvested to evaluate the efficiency of IGF-IR knockdown (top left panels). PC3-Ch1 and PC3-Ch2 cells were starved in serum-free medium for 24 h and where indicated, induced with 75 μM ZnSO_4_ for additional 6 h to promote the expression of chimeric proteins. Cells were harvested for analysis of β_1_ subunit expression (top right panels). A parallel set of samples was processed to confirm the inducible expression of the chimeras by FACS analysis using an Ab to CD4 (lower panels). 10,000 cells in each sample were acquired and data are shown in histograms with the x-axis representing mean relative CD4 expression and the y-axis representing the number of cells. (B) PC3-Ch1 and PC3-Chβ_1_C (control cells expressing extracellular domain of murine CD4 and the transmembrane and cytoplasmic domains of the β_1_C) incubated with 75 μM ZnSO_4_ to induce the expression of the cytoplasmic domain of β_1_A or β_1_C integrins, respectively. Lysates were immunoprecipitated with either control IgG or Ab against chimeric CD4 domains. Immunocomplexes were analyzed for IGF-IR expression by immunoblotting. Input lysates were run as controls.

To explore whether the exogenous expression of the cytoplasmic domain of β_1_A alters IGF-IR-mediated regulation of endogenous β_1_ integrin subunits, chimeric constructs composed of transmembrane and cytoplasmic domain of β_1_A integrin with CD4 extracellular domain (Ch1) were used. The cytoplasmic domain of the β_1_ subunit exists in five different spliced forms; the most widely expressed form in cancer, β_1_A, regulates β_1_ localization, cell proliferation and migration [2]. We speculated that binding of the cytoplasmic domain of β_1_ integrins to the IGF-IR would lead to some competition for binding of IGF-IR to different forms of β_1_ and result in a β_1_ protective effect under depleted IGF-IR conditions. Expression of the Ch1 chimera (Ch1 cells), or Ch2 chimera, which corresponds to the transmembrane domain of β_1_ plus the CD4 extracellular domain (Ch2 cells), in PC3 cells was induced by ZnSO_4_ treatment. IGF-IR depletion in stably transfected cells was confirmed by immunoblot analysis (Fig. 4A, top left panel). Induction of the cytoplasmic β_1_ variant was confirmed by FACS (Fig. 4A, lower panels). Upon induction, IGF-IR would be expected to redistribute and bind to both the endogenous β_1_ and exogenous cytoplasmic variant. Exogenous induction of the β_1_ cytoplasmic domain, however, does not alter the IGF-IR-mediated regulation of endogenous β_1_ integrin levels (Fig 4A, top right panel). In order to investigate if the β_1_A cytoplasmic variant physically interacts with the IGF-IR, immunoprecipitation of CD4 in PC3-Ch1 and PC3-Ch β_1_C cells (stably transfected with cytoplasmic domain of β_1_C integrin plus the CD4 extracellular domain [29]) was carried out after incubating the cells with ZnSO_4_. The immunoblot shows (upper panel) the presence of IGF-IR in the cell lysate but not in CD4 immunoprecipitated samples. The lower panel shows that CD4 was efficiently immunoprecipitated; an irrelevant band was also detected in the IgG immunoprecipitated samples. The data indicate that the cytoplasmic domain of the β_1_A integrin variant does not interact with endogenous IGF-IR (Fig. 4B).

### Enhanced proteasomal degradation of β_1_ integrin subunits in the absence of IGF-IR

**Figure 5:**
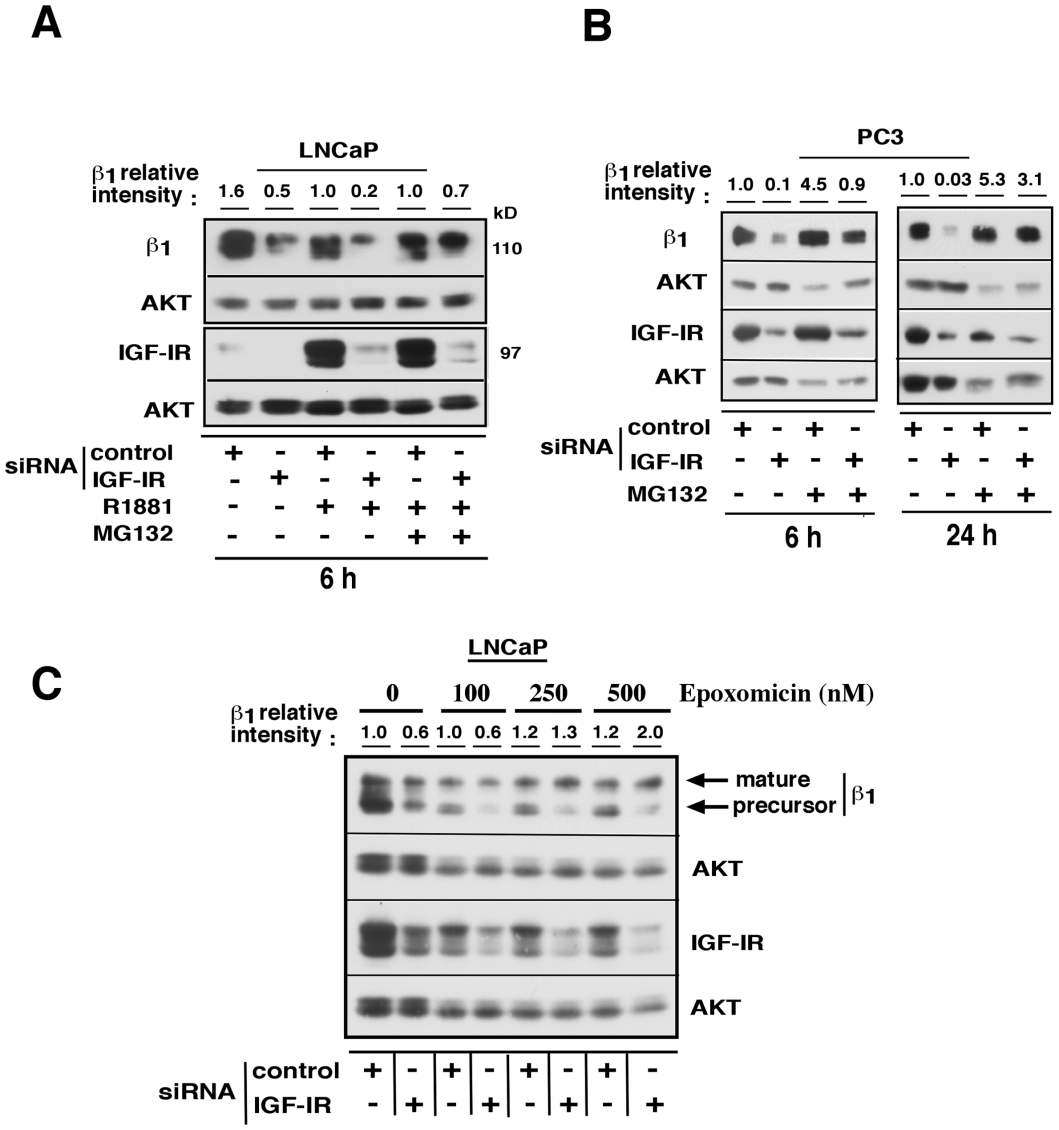
Loss of IGF-IR causes proteasome-mediated degradation of β_1_ integrins. (A) LNCaP cells were transfected with either control or IGF-IR siRNA. Twenty four h later, cells were grown in medium containing 2% CSS for additional 24 h and treated with vehicle, 1 nM R1881 and/or 10 μM MG132 for 6 h. Cell lysates were analyzed for expression of β_1_ integrin subunit and IGF-IR by immunoblotting. AKT was used as a loading control. The band intensities of the β_1_ integrin subunit were quantitated by ImageJ analysis and normalized with those of AKT as a loading control. The relative intensity values are expressed as percent of the control sample transfected with control siRNA alone. (B) PC3 cells were transfected with either control or IGF-IR siRNA. Twenty four h after transfection, cells were treated with 10 μM MG132 for 6 or 24 h in RPMI medium containing 10% serum. Cell lysates were analyzed for expression of β_1_ integrin subunit and IGF-IR by immunoblotting. AKT was used as a loading control. β_1_ integrin subunits were quantitated by ImageJ as above and values normalized with those of loading control AKT. Relative intensity values of β_1_ are expressed as percent of the control sample transfected with control siRNA alone. (C) LNCaP cells were transfected as above and 24 h later cells were cultured in medium containing 2% CSS for additional 24 h followed by treatment with 0, 100, 250 or 500 nM epoxomicin in combination with 1 nM R1881 for 18 h. Cell lysates were analyzed for mature and precursor forms of β_1_ integrin subunit and IGF-IR by immunoblotting. AKT serves as a loading control. Relative band intensities of mature β_1_ integrin were determined by ImageJ analysis as above.

After demonstrating that IGF-IR does not regulate β_1_ integrin transcripts, we sought to determine whether IGF-IR-mediated regulation of β_1_ integrin levels would occur at post-translational level. Transient depletion of IGF-IR in LNCaP cells was followed by R1881 treatment alone or in combination with a proteasome inhibitor, MG132, for 6 h. R1881 was used to enhance the basal expression levels of IGF-IR and β_1_ integrin subunit as reported by our group earlier [23] and cell lysates were analyzed. The reduction of β_1_ integrin levels induced by IGF-IR depletion was abolished upon cell treatment with this proteasome inhibitor (Fig. 5A). The results of this experiment were confirmed in PC3 cells after transfection with either control siRNA or IGF-IR siRNA followed by treatment with MG132 for either 6 or 24 h (Fig. 5B). We additionally corroborated these results by using different doses of epoxomicin, another highly specific proteasome inhibitor [30]. LNCaP cells were transfected with either control or IGF-IR siRNA as above and treated with increasing concentrations of epoxomicin. β_1_ integrin subunits are present in either precursor or mature forms (110 and 130 kD respectively) and both are downregulated upon IGF-IR loss. The data show that epoxomicin blocks the degradation of the mature form of the β_1_ integrin subunit (Fig. 5C). The mature β_1_ receptor alone appears to follow proteasomal degradation after internalization. The precursor form of β_1_ (110 kD) which needs further post-translational modifications to undergo maturation is not recovered by proteasomal inhibition. The data show that IGF-IR stabilizes β_1_ integrin subunit expression by inhibiting its proteasomal degradation.

### Analysis of α integrin subunits upon IGF-IR downregulation

**Figure 6:**
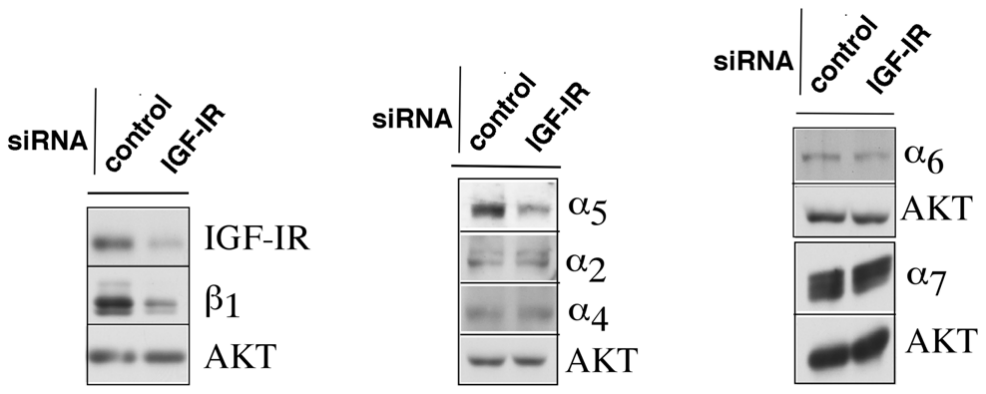
β_1_ integrin downregulation upon IGF-IR depletion is associated with reduced α_5_ integrin subunit expression. LNCaP cells were transfected with either control or IGF-IR siRNA and treated with 2% CSS-containing medium for 24 h followed by treatment with 1 nM R1881 for 24 h. IGF-IR and β1 integrin downregulation was evaluated by immunoblots. The lysates were then analyzed for the expression of various α integrin subunits. Specific Abs against α_2_, α_4_, α_5_, α_6_ and α_7_ integrin subunits were used to identify the α integrin partner of the β_1_ integrin subunit, which is downregulated upon IGF-IR depletion.

Integrins are heterodimers consisting of α and β subunits. There are 24 possible heterodimers with the ability to activate specific signaling pathways [19]. β_1_ integrins, among other subunits, are known to heterodimerize with α2, α3, α4, α5, α6 and α7 integrin subunits, which are expressed in LNCaP cells. Since reduction of IGF-IR expression levels leads to the downregulation of the β_1_ integrin subunit, we decided to determine which α integrin subunit was affected by IGF-IR downregulation. We demonstrate a significant reduction of the α5 integrin subunit in conjunction with reduced IGF-IR and β_1_ levels (Fig. 6). No change was detected in the expression levels of other α integrin heterodimeric partners (Fig. 6 and data not shown). These results are consistent with our previous observations in PC3 cells where abrogation of β_1_ integrins by shRNA led to a significant reduction in the surface expression of α5 integrin subunit [3]. Our data demonstrate that the major complex regulated by IGF-IR is α5β_1_ integrin.

## Discussion

This study describes a novel observation that IGF-IR functions in PrCa cells are partially mediated by β_1_ integrins. To dissect the mechanism by which IGF-IR regulates β_1_ integrin expression, we demonstrate that IGF-IR enhances β_1_ integrin stability by reducing its proteasomal degradation. We also show that the α_5_ integrin subunit associated with β_1_ is selectively downregulated upon IGF-IR loss.

Substantial epidemiological and preclinical data have identified the IGF-IR pathway as an important regulator of tumor cell biology. The disappointing results, however, from several clinical trials aimed to inhibit IGF-IR, are prompting researchers to develop predictive biomarkers to improve patient selection that would benefit from therapies targeting IGF-IR. Moreover, a clearer understanding of relative proportions of IGF-IR and IR complexes in tumors is necessary. In this regard, tyrosine kinase inhibitors specific for both the IGF-IR and IR could address the concern that increased IR signaling occurs upon IGF-IR inhibition [31]. Interestingly, a combination of IGF-IR and MEK inhibitors was recently reported to result in significant inhibition of K-Ras-mutant lung cancer lines and also improve effectiveness in two mouse models of K-Ras-driven lung cancer [32]. Understanding the functional crosstalk of IGF-IR with integrins will potentially open up novel approaches to block this crucial signaling pathway. Our demonstration of the critical role played by IGF-IR and β_1_ in regulating biological responses of PrCa cells, both in anchorage-dependent and -independent growth, is in agreement with previous findings that integrins are crucial for IGF-IR-mediated mitogenic and transforming activities [10], [33], [34], [35]. Similar crosstalk has been observed between IGF-IR and E-cadherin, a complex shown to mediate cell-cell adhesion in human breast cancer cells [36], and among IGF-IR, E-cadherin and α_V_ integrins, shown to have dynamic interactions under the control of α catenin [37]. The stimulatory, as described here, or inhibitory, as described for TNF receptor-1, effect of the crosstalk between growth factor receptors and cell-surface integrins is an area which has attracted much interest in recent years [38], [39], [40], [41]. The crosstalk appears to be mediated by a direct interaction between growth factor receptors and cell-surface integrins; we have previously demonstrated that β_1_ integrins physically associate with the IGF-IR [12]. In an effort to further characterize this interaction, we exogenously induced a chimeric protein containing the cytoplasmic domain of the β_1_ integrin subunit to test if it binds IGF-IR. We do not, however, observe any association of the chimeric cytoplasmic domain of β_1_ integrins with IGF-IR; thus, further analysis is necessary to identify the domains that mediate this interaction. It should be stressed that IGF-1 has been reported to directly bind to integrins and induce the formation of a ternary complex containing integrin-IGF1-IGF-IR [42], [43]. The authors report that an integrin-binding-defective mutant of IGF-1 (R36E/R37E IGF-1), which still binds IGF-IR, acts as a dominant-negative antagonist of IGF-IR and suppresses tumorigenesis; they also show that IGF-1 binds to α_6_β_4_ as well as to β_1_ integrins, consistent with our data.

IGF-IR regulation of β_1_ integrin expression, is critical in the context of reported alterations in the IGF-1 axis signaling and expression during cancer progression [44], and implies that variations of the levels of one receptor may influence the profile of other receptors. Consistent with this, we have reported concurrent upregulation of β_1_ integrins and IGF-IR in prostatic intraepithelial neoplasia and well differentiated prostate carcinoma [10]. We now demonstrate that in the absence of IGF-IR, β_1_ integrins are subjected to proteasome-mediated degradation suggesting that the interaction between IGF-IR and β_1_ integrins not only provides synergistic signaling but enhances the stability of both proteins. β_1_ loss by proteasomal degradation has been previously reported in *Talin-1* null embryonic stem cells leading to defective integrin-adhesion complex assembly. Since Talin-1 overexpression has been reported to enhance PrCa invasion and disrupting Talin-1 signaling/focal adhesion interactions was proposed to have a therapeutic significance in targeting metastatic PrCa [45], [46], we speculate that the IGF-IR/β_1_ integrin pathway may be altered in the absence of Talin-1. IGF-IR mutants with impaired PI3K-AKT signaling were reported to exhibit receptor ubiquitination and were degraded by proteasomes. However, C-terminal truncated IGF-IR failed to undergo ubiquitination and was exclusively degraded through lysosomal pathways [47]. Moreover, our experiments suggesting proteasomal degradation of β_1_ upon IGF-IR depletion do not exclude the possibility that β_1_ integrins may also be in part processed through lysosomal or recycled through endosomal pathways. Similar to what is known of the ligand-induced ubiquitination of growth factor receptors, α_5_β_1_ was recently reported to be ubiquitinated followed by degradation in response to fibronectin binding [48]. Our results suggest that in the absence of IGF-IR, β_1_ integrins are ubiquitinated and marked for proteasomal and/or lysosomal degradation. A Sorting Nexin family protein, SNX17 was recently reported to regulate the stability of β_1_ and it would be crucial to determine if SNX17 is involved in IGF-IR-mediated regulation of β_1_ integrins [49], [50]. Furthermore, it is equally important to investigate if β_1_-integrin degradation triggered by IGF-IR loss involves clathrin- or caveolin-dependent endocytosis. It could be speculated that recycling of β_1_ from early endosomes back to cell membrane can occur through a rapid recycling route by returning to the cell surface directly from endosomes or through a slow recycling route involving Rab GTPases such as Rab4 and Rab11 [28], [51].

Our data show that proteasomal inhibition rescues the degradation of the β_1_ integrin subunit upon IGF-IR downregulation. Although we have observed the downregulation of both mature (130 kD) and precursor (110 kD) forms of β_1_ integrins upon IGF-IR knockdown, only the mature β_1_ form is recognized by the proteasomal machinery and thus preferentially degraded. It is, however, not clear how IGF-IR regulates the immature form of β_1_ integrins. Since we did not observe any changes in mRNA levels upon ablation of IGF-IR, other effectors downstream of IGF-IR might be involved in regulating the immature form of β_1_ integrins.

Downregulation of the β_1_ integrin subunit has been shown to significantly reduce the surface expression of the associated α_5_ subunit in PrCa cell lines [3]. Our novel finding that α_5_ integrin subunit is significantly reduced upon IGF-IR downregulation, is consistent with direct causal role of IGF-IR on β_1_ expression, as we previously reported [24]. Consistent with this, other laboratories have recently shown that *in vivo* inhibition of α_5_ integrin significantly reduces tumor growth [52]. These findings support a crucial role for the α_5_ subunit and suggest that inhibitors of α_5_ integrin may be useful in blocking tumor progression. In this regard, it is worth mentioning that miR-92a was demonstrated to inhibit peritoneal dissemination of ovarian cancer cells by inhibiting α_5_ expression, which was accompanied by the inhibition of cancer cell adhesion, invasion and proliferation [53]. α_5_β_1_ was also observed to simultaneously control EGFR-dependent proliferation and Akt-dependent pro-survival signaling in epidermoid carcinoma cells [54]. Furthermore, FGFR2-mediated osteoblast detachment and apoptosis was reported to be caused by Cbl-dependent ubiquitination of α_5_ integrin [55]. Endosomal accumulation of integrins is prevented by ligand-mediated degradation of the α_5_β_1_ integrin, which might otherwise develop non-productive adhesion sites. Fibroblast migration was reported to be regulated by trafficking of fibronectin and ubiquitinated α_5_β_1_ complexes to lysosomes for degradation [48]. It could be speculated that upon loss of IGF-IR, α_5_β_1_ integrin is shuttled to proteasomes and lysosomes for degradation instead of being translocated to early endosomes for recycling.

In conclusion, this paper highlights a novel pathway mediated by IGF-IR and α_5_β_1_ integrin in PrCa growth and dissects the mechanism by which IGF-IR regulates the expression of α_5_β_1_ integrin. We propose that IGF-IR signaling, by controlling the stability of the α_5_β_1_ integrin through a proteasomal pathway, tightly regulates pro-survival signaling in PrCa.
